# Dichlorido[2,4-dimethyl-*N*-(pyridin-2-yl­methyl­idene)aniline-κ^2^ *N*,*N*′]dimethyl­tin(IV)

**DOI:** 10.1107/S1600536811010439

**Published:** 2011-03-26

**Authors:** Sedigheh Loni, Mohamad Reza Talei Bavil Olyai, Fatemeh Roodbari, Behrouz Notash

**Affiliations:** a Department of Chemistry, Islamic Azad University, Karaj Branch, Karaj, Iran; b Department of Chemistry, Islamic Azad University, South Tehran Branch, Tehran, Iran; c Department of Chemistry, Shahid Beheshti University, G. C., Evin, Tehran 1983963113, Iran

## Abstract

The asymmetric unit of the title compound, [Sn(CH_3_)_2_Cl_2_(C_14_H_14_N_2_)], contains two crystallographically independent mol­ecules. In each mol­ecule, the Sn^IV^ atom is six-coordinated in a distorted octa­hedral geometry by one bidentate 2,4-di­methyl-*N*-(pyridin-2-yl­methyl­idene)aniline ligand, two methyl groups and two Cl atoms. In the crystal, inter­molecular C—H⋯Cl hydrogen bonds link the mol­ecules. There are π–π contacts between the pyridine rings of the ligands [centroid–centroid distance = 3.761 (4) Å].

## Related literature

For applications of Schiff bases and their metal complexes, see: Azza & Abu (2006 ▶); Dudek & Dudek (1966 ▶); McAuliffe *et al.* (1986 ▶); Mladenova *et al.* (2002 ▶); Pandeya *et al.* (1999 ▶); Panneerselvam *et al.* (2005 ▶); Papić *et al.* (1994 ▶); Singh *et al.* (2006 ▶); Sridhar *et al.* (2001 ▶); Vlcek (2002 ▶); Walsh *et al.* (1996 ▶). For related structures, see: Ali *et al.* (2004 ▶); Fallah Nejad *et al.* (2010 ▶); Labisbal *et al.* (2006 ▶); Talei Bavil Olyai *et al.* (2008 ▶, 2010*a* ▶,*b* ▶).
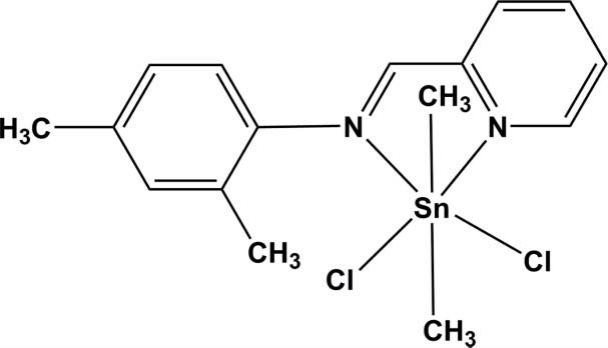


## Experimental

### 

#### Crystal data

[Sn(CH_3_)_2_Cl_2_(C_14_H_14_N_2_)]
*M* *_r_* = 429.95
Orthorhombic, 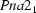

*a* = 15.507 (3) Å
*b* = 7.3500 (15) Å
*c* = 32.175 (6) Å
*V* = 3667.2 (12) Å^3^
*Z* = 8
Mo *K*α radiation
μ = 1.68 mm^−1^
*T* = 298 K
0.30 × 0.28 × 0.20 mm

#### Data collection

Stoe IPDS-2 diffractometer
Absorption correction: numerical (*X-SHAPE* and *X-RED32*; Stoe & Cie, 2005 ▶) *T*_min_ = 0.607, *T*_max_ = 0.711
25085 measured reflections
9838 independent reflections
7280 reflections with *I* > 2σ(*I*)
*R*_int_ = 0.080

#### Refinement

*R*[*F* ^2^ > 2σ(*F* ^2^)] = 0.038
*wR*(*F* ^2^) = 0.116
*S* = 0.97
9838 reflections
387 parameters
1 restraint
H-atom parameters constrained
Δρ_max_ = 0.75 e Å^−3^
Δρ_min_ = −0.56 e Å^−3^
Absolute structure: Flack (1983 ▶), 4819 Friedel pairs
Flack parameter: 0.19 (3)

### 

Data collection: *X-AREA* (Stoe & Cie, 2005 ▶); cell refinement: *X-AREA*; data reduction: *X-AREA*; program(s) used to solve structure: *SHELXS97* (Sheldrick, 2008 ▶); program(s) used to refine structure: *SHELXL97* (Sheldrick, 2008 ▶); molecular graphics: *ORTEP-3* (Farrugia, 1997 ▶); software used to prepare material for publication: *WinGX* (Farrugia, 1999 ▶).

## Supplementary Material

Crystal structure: contains datablocks I, global. DOI: 10.1107/S1600536811010439/hy2416sup1.cif

Structure factors: contains datablocks I. DOI: 10.1107/S1600536811010439/hy2416Isup2.hkl

Additional supplementary materials:  crystallographic information; 3D view; checkCIF report

## Figures and Tables

**Table 1:** Selected bond lengths (Å)

Sn1—C15	2.126 (8)
Sn1—C16	2.118 (7)
Sn1—N1	2.470 (5)
Sn1—N2	2.468 (8)
Sn1—Cl1	2.5213 (19)
Sn1—Cl2	2.4859 (19)
Sn2—C31	2.124 (7)
Sn2—C32	2.130 (7)
Sn2—N3	2.456 (5)
Sn2—N4	2.449 (8)
Sn2—Cl3	2.4908 (19)
Sn2—Cl4	2.5170 (19)

**Table 2:** Hydrogen-bond geometry (Å, °)

*D*—H⋯*A*	*D*—H	H⋯*A*	*D*⋯*A*	*D*—H⋯*A*
C9—H9⋯Cl2^i^	0.93	2.73	3.608 (6)	157
C25—H25⋯Cl3^ii^	0.93	2.70	3.570 (7)	155

Symmetry codes: (i) 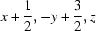; (ii) 
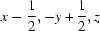
.

**Fig. 1.**
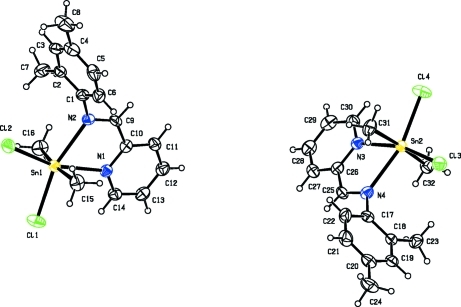
The molecular structure of the title compound. Displacement ellipsoids are drawn at the 30% probability level.

**Fig. 2.**
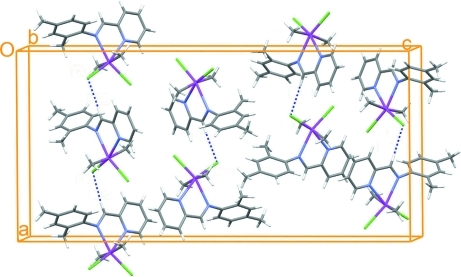
The packing diagram of the title compound. Intermolecular C—H···Cl hydrogen bonds are shown as blue dashed lines.

**Fig. 3.**
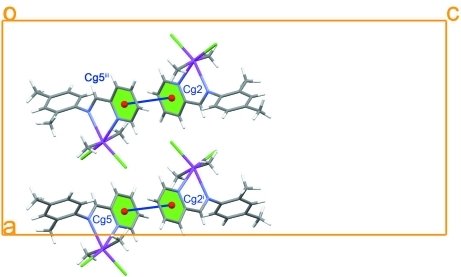
The packing diagram of the title compound, showing π–π interactions. Cg2 and Cg5 are the centroids of N1, C10–C14 and N3, C26–C30 rings, respectively. [Symmetry codes: (i) 1/2+x, 3/2-y, z; (iii) -1/2+x, 3/2-y, z.]

## supplementary crystallographic information

### Comment

Compounds with an azomethine group, C═N, are known as Schiff bases, which are usually synthesized from the condensation of primary amines and active carbonyl groups. The Schiff bases and their metal complexes are an important class of compounds in medicinal and pharmaceutical field. They show biological applications including antibacterial (Azza & Abu, 2006; Dudek & Dudek, 1966), antifungal (Pandeya *et al.*, 1999; Panneerselvam *et al.*, 2005; Singh *et al.*, 2006; Sridhar *et al.* 2001) and antitumor activities (Mladenova *et al.*, 2002; Walsh *et al.*, 1996) and industrial uses, especially in catalysis (McAuliffe *et al.*, 1986), dying (Papić *et al.*, 1994), electronic and optic (Vlcek, 2002). In our ongoing studies on the synthesis and structural determination of transition metal complexes with iminopyridine ligands (Fallah Nejad *et al.*, 2010; Talei Bavil Olyai *et al.*, 2008, 2010*a*,b), we report here the crystal structure of the title compound, derived from the Schiff base ligand, 2,4-dimethyl-*N*-(pyridin-2-ylmethylene)aniline.

The title compound consists of two crystallographically independent molecules in the asymmetric unit, both with a similar six-coordinated environment. The Sn^IV^ atom is surrounded by two (one imino and one pyridine) N atoms belonging to the bidentate chelating iminopyridine ligand, two methyl groups and two Cl atoms (Fig. 1). The Sn—N, Sn—C and Sn—Cl bond distances (Table 1) are within normal ranges, which are similar to those reported in literature (Ali *et al.*, 2004; Labisbal *et al.*, 2006). The bond lengths and angles around the Sn^IV^ atoms show deviation from an ideal octahedral geometry. The Sn1—N2 and Sn2—N4 imine distances [2.468 (8) and 2.449 (8) Å] are approximately close to the Sn1—N1 and Sn2—N3 pyridine distances [2.470 (5) and 2.456 (5) Å]. The N2—C9 and N4—C25 bond lengths of 1.254 (10) and 1.277 (10) Å are typical for the C═N double bond.

In the crystal, intermolecular C—H···Cl hydrogen bonds (Table 2, Fig. 2) link the molecules, which may be effective in the stabilization of the structure. π–π contacts between the pyridine rings, *Cg*2^i^···*Cg*5 [*Cg*2 and *Cg*5 are the centroids of N1, C10—C14 and N3, C26—C30 rings, respectively; symmetry code: (i) 1/2+*x*, 3/2-*y*, *z*], further stabilize the structure, with centroid–centroid distance of 3.761 (4) Å (Fig. 3).

### Experimental

For the preparation of the title compound, a solution of 2,4-dimethyl-*N*-(pyridin-2-ylmethylene)aniline (0.210 g, 1 mmol) in ethanol (10 ml) was added to a solution of Sn(CH_3_)_2_Cl_2_ (0.220 g, 1 mmol) in methanol (10 ml). The resulting colorless solution was stirred for 2 hra at room temperature and then left to evaporate slowly at 3–5°C. After two weeks, yellow crystals of the title compound were isolated (yield: 0.329 g, 76.4%; m.p.: 416–419 K).

### Refinement

H atoms were positioned geometrically and refined as riding atoms, with C—H = 0.93 (CH) and 0.96 (CH_3_) Å and with *U*_iso_(H) = 1.2(1.5 for methyl)*U*_eq_(C).

### Crystal data

**Table:**

[Sn(CH_3_)_2_Cl_2_(C_14_H_14_N_2_)]	*F*(000) = 1712.0
*M**_r_* = 429.95	*D*_x_ = 1.558 Mg m^−^^3^
Orthorhombic, *P**n**a*2_1_	Mo *K*α radiation, λ = 0.71073 Å
Hall symbol: P 2c -2n	Cell parameters from 9838 reflections
*a* = 15.507 (3) Å	θ = 2.5–29.2°
*b* = 7.3500 (15) Å	µ = 1.68 mm^−^^1^
*c* = 32.175 (6) Å	*T* = 298 K
*V* = 3667.2 (12) Å^3^	Block, yellow
*Z* = 8	0.30 × 0.28 × 0.20 mm

### Data collection

**Table:**

Stoe IPDS-2 diffractometer	9838 independent reflections
Radiation source: fine-focus sealed tube	7280 reflections with *I* > 2σ(*I*)
graphite	*R*_int_ = 0.080
ω scans	θ_max_ = 29.2°, θ_min_ = 2.5°
Absorption correction: numerical (*X-SHAPE* and *X-RED32*; Stoe & Cie, 2005)	*h* = −18→21
*T*_min_ = 0.607, *T*_max_ = 0.711	*k* = −10→9
25085 measured reflections	*l* = −44→44

### Refinement

**Table:**

Refinement on *F*^2^	Secondary atom site location: difference Fourier map
Least-squares matrix: full	Hydrogen site location: inferred from neighbouring sites
*R*[*F*^2^ > 2σ(*F*^2^)] = 0.038	H-atom parameters constrained
*wR*(*F*^2^) = 0.116	*w* = 1/[σ^2^(*F*_o_^2^) + (0.0637*P*)^2^] where *P* = (*F*_o_^2^ + 2*F*_c_^2^)/3
*S* = 0.97	(Δ/σ)_max_ = 0.004
9838 reflections	Δρ_max_ = 0.75 e Å^−^^3^
387 parameters	Δρ_min_ = −0.56 e Å^−^^3^
1 restraint	Absolute structure: Flack (1983), 4819 Friedel pairs
Primary atom site location: structure-invariant direct methods	Flack parameter: 0.19 (3)

### Fractional atomic coordinates and isotropic or equivalent isotropic displacement parameters (Å^2^)

**Table:**

	*x*	*y*	*z*	*U*_iso_*/*U*_eq_	
Sn1	0.18692 (2)	0.80347 (6)	0.431585 (9)	0.04196 (9)	
N1	0.2970 (3)	0.9910 (7)	0.39634 (15)	0.0452 (11)	
C10	0.3773 (4)	0.9827 (8)	0.41197 (18)	0.0448 (13)	
C13	0.3446 (7)	1.2245 (11)	0.3505 (3)	0.056 (2)	
H13	0.3318	1.3058	0.3292	0.067*	
C4	0.3997 (5)	0.3521 (12)	0.5478 (3)	0.069 (2)	
C11	0.4436 (4)	1.0900 (10)	0.3980 (2)	0.0545 (15)	
H11	0.4988	1.0793	0.4091	0.065*	
C7	0.2816 (7)	0.8040 (15)	0.5495 (3)	0.072 (2)	
H7A	0.2225	0.7945	0.5408	0.107*	
H7B	0.3064	0.9132	0.5384	0.107*	
H7C	0.2840	0.8077	0.5793	0.107*	
C6	0.3986 (5)	0.4761 (10)	0.4793 (2)	0.0574 (17)	
H6	0.4134	0.4655	0.4514	0.069*	
C1	0.3542 (4)	0.6262 (9)	0.49281 (18)	0.0467 (13)	
C2	0.3302 (4)	0.6450 (10)	0.53432 (19)	0.0478 (14)	
C3	0.3556 (5)	0.5078 (11)	0.5611 (2)	0.0574 (17)	
H3	0.3428	0.5196	0.5892	0.069*	
C12	0.4258 (6)	1.2161 (11)	0.3666 (3)	0.0568 (19)	
H12	0.4688	1.2933	0.3568	0.068*	
C14	0.2824 (4)	1.1118 (9)	0.36599 (18)	0.0484 (14)	
H14	0.2271	1.1194	0.3549	0.058*	
C5	0.4213 (5)	0.3404 (11)	0.5069 (3)	0.0646 (19)	
H5	0.4519	0.2398	0.4974	0.078*	
Sn2	1.06671 (2)	0.29828 (6)	0.226572 (9)	0.04186 (10)	
N4	0.9250 (5)	0.2614 (8)	0.1941 (3)	0.0489 (17)	
C22	0.8552 (5)	−0.0265 (11)	0.1770 (2)	0.0622 (19)	
H22	0.8388	−0.0373	0.2047	0.075*	
C29	0.9057 (8)	0.7210 (11)	0.3062 (3)	0.062 (2)	
H29	0.9171	0.8027	0.3275	0.075*	
C25	0.8622 (4)	0.3456 (9)	0.21111 (19)	0.0470 (14)	
H25	0.8062	0.3227	0.2022	0.056*	
C26	0.8770 (4)	0.4778 (9)	0.24446 (18)	0.0481 (14)	
C17	0.9013 (4)	0.1215 (9)	0.16414 (18)	0.0452 (13)	
C18	0.9281 (4)	0.1373 (9)	0.12304 (18)	0.0434 (12)	
C21	0.8328 (5)	−0.1620 (11)	0.1484 (3)	0.068 (2)	
H21	0.8007	−0.2620	0.1571	0.081*	
C23	0.9785 (6)	0.2992 (12)	0.1078 (3)	0.0597 (17)	
H23A	1.0367	0.2918	0.1178	0.090*	
H23B	0.9522	0.4089	0.1179	0.090*	
H23C	0.9787	0.3001	0.0779	0.090*	
C19	0.9069 (4)	0.0013 (10)	0.09576 (19)	0.0524 (16)	
H19	0.9259	0.0091	0.0684	0.063*	
C28	0.8256 (6)	0.7091 (12)	0.2895 (3)	0.061 (2)	
H28	0.7815	0.7838	0.2990	0.074*	
C24	0.8344 (7)	−0.2936 (14)	0.0771 (4)	0.079 (3)	
H24A	0.8789	−0.3031	0.0565	0.119*	
H24B	0.7808	−0.2629	0.0639	0.119*	
H24C	0.8285	−0.4078	0.0913	0.119*	
C27	0.8106 (4)	0.5860 (11)	0.2585 (2)	0.0570 (16)	
H27	0.7559	0.5754	0.2469	0.068*	
C20	0.8578 (5)	−0.1481 (11)	0.1078 (2)	0.0584 (17)	
C31	1.0238 (6)	0.0794 (11)	0.2644 (2)	0.068 (2)	
H31A	0.9768	0.1196	0.2815	0.102*	
H31B	1.0703	0.0386	0.2817	0.102*	
H31C	1.0048	−0.0189	0.2470	0.102*	
N2	0.3306 (5)	0.7655 (8)	0.4634 (2)	0.0436 (15)	
N3	0.9570 (3)	0.4873 (7)	0.26072 (15)	0.0449 (11)	
C32	1.0925 (5)	0.5382 (11)	0.1916 (2)	0.0621 (19)	
H32A	1.0402	0.5801	0.1788	0.093*	
H32B	1.1344	0.5116	0.1705	0.093*	
H32C	1.1146	0.6308	0.2097	0.093*	
C15	0.2288 (5)	0.5808 (12)	0.3946 (3)	0.067 (2)	
H15A	0.2808	0.6136	0.3802	0.100*	
H15B	0.1849	0.5508	0.3747	0.100*	
H15C	0.2396	0.4776	0.4121	0.100*	
Cl1	0.06818 (14)	0.8806 (4)	0.38104 (8)	0.0797 (6)	
Cl2	0.11045 (12)	0.5992 (3)	0.48092 (6)	0.0696 (5)	
C9	0.3909 (4)	0.8486 (10)	0.44574 (18)	0.0474 (14)	
H9	0.4471	0.8249	0.4543	0.057*	
Cl3	1.14422 (12)	0.0871 (3)	0.17892 (6)	0.0660 (5)	
Cl4	1.18382 (14)	0.3783 (4)	0.27756 (8)	0.0805 (7)	
C30	0.9704 (4)	0.6088 (10)	0.2907 (2)	0.0519 (15)	
H30	1.0256	0.6192	0.3018	0.062*	
C8	0.4216 (8)	0.2040 (17)	0.5793 (4)	0.104 (5)	
H8A	0.3695	0.1456	0.5883	0.156*	
H8B	0.4503	0.2573	0.6027	0.156*	
H8C	0.4588	0.1156	0.5665	0.156*	
C16	0.1653 (6)	1.0461 (11)	0.4656 (2)	0.068 (2)	
H16A	0.2131	1.0675	0.4839	0.102*	
H16B	0.1133	1.0343	0.4815	0.102*	
H16C	0.1597	1.1464	0.4466	0.102*	

### Atomic displacement parameters (Å^2^)

**Table:**

	*U*^11^	*U*^22^	*U*^33^	*U*^12^	*U*^13^	*U*^23^
Sn1	0.03716 (19)	0.0435 (2)	0.0452 (2)	0.00291 (18)	−0.00340 (15)	0.0017 (3)
N1	0.049 (3)	0.044 (3)	0.043 (2)	0.004 (2)	0.002 (2)	0.005 (2)
C10	0.051 (3)	0.039 (3)	0.045 (3)	0.003 (3)	0.004 (3)	0.001 (2)
C13	0.073 (5)	0.046 (4)	0.048 (4)	0.005 (4)	0.004 (4)	0.005 (3)
C4	0.061 (4)	0.065 (5)	0.080 (5)	−0.021 (4)	−0.023 (4)	0.033 (4)
C11	0.045 (3)	0.062 (4)	0.056 (3)	−0.011 (3)	−0.004 (3)	0.004 (3)
C7	0.079 (6)	0.089 (6)	0.047 (4)	0.007 (5)	−0.001 (4)	−0.009 (5)
C6	0.049 (4)	0.064 (5)	0.059 (4)	0.012 (3)	0.006 (3)	0.009 (3)
C1	0.047 (3)	0.047 (3)	0.046 (3)	0.005 (3)	−0.008 (2)	0.010 (3)
C2	0.040 (3)	0.055 (3)	0.048 (3)	−0.007 (3)	−0.007 (3)	0.005 (3)
C3	0.050 (4)	0.071 (5)	0.051 (3)	−0.014 (3)	−0.016 (3)	0.019 (3)
C12	0.066 (5)	0.051 (4)	0.053 (4)	−0.014 (4)	0.004 (4)	0.007 (3)
C14	0.050 (3)	0.045 (4)	0.050 (3)	0.009 (3)	−0.006 (3)	0.007 (3)
C5	0.045 (4)	0.056 (4)	0.093 (5)	0.009 (3)	0.004 (4)	0.016 (4)
Sn2	0.0385 (2)	0.0460 (2)	0.04104 (19)	−0.00384 (18)	−0.00327 (16)	0.0047 (3)
N4	0.059 (4)	0.046 (3)	0.042 (4)	−0.006 (2)	0.003 (3)	−0.002 (2)
C22	0.056 (4)	0.071 (5)	0.060 (4)	−0.022 (4)	0.011 (3)	−0.005 (3)
C29	0.099 (6)	0.036 (3)	0.052 (4)	−0.011 (4)	0.004 (4)	−0.006 (3)
C25	0.032 (3)	0.055 (4)	0.054 (3)	−0.003 (3)	−0.005 (2)	−0.006 (3)
C26	0.054 (4)	0.048 (4)	0.043 (3)	−0.003 (3)	0.003 (3)	−0.001 (3)
C17	0.041 (3)	0.046 (3)	0.049 (3)	−0.002 (3)	−0.010 (3)	−0.006 (3)
C18	0.035 (3)	0.051 (3)	0.044 (3)	0.001 (3)	−0.003 (2)	0.003 (2)
C21	0.061 (4)	0.060 (5)	0.083 (5)	−0.023 (4)	0.003 (4)	−0.008 (4)
C23	0.068 (5)	0.059 (4)	0.052 (4)	0.001 (4)	0.004 (3)	0.014 (4)
C19	0.040 (3)	0.068 (5)	0.049 (3)	0.008 (3)	−0.007 (2)	−0.009 (3)
C28	0.073 (5)	0.051 (4)	0.061 (4)	0.009 (5)	0.009 (4)	−0.005 (4)
C24	0.064 (5)	0.080 (6)	0.093 (7)	−0.001 (5)	−0.028 (5)	−0.028 (6)
C27	0.043 (3)	0.069 (5)	0.060 (4)	0.000 (3)	−0.002 (3)	−0.003 (3)
C20	0.049 (4)	0.052 (4)	0.075 (4)	0.001 (3)	−0.014 (3)	−0.012 (3)
C31	0.081 (5)	0.057 (4)	0.066 (4)	−0.006 (4)	0.019 (4)	0.022 (4)
N2	0.047 (3)	0.044 (3)	0.040 (4)	0.003 (2)	−0.004 (3)	0.006 (2)
N3	0.050 (3)	0.044 (3)	0.040 (2)	−0.003 (2)	0.000 (2)	−0.002 (2)
C32	0.056 (4)	0.058 (4)	0.072 (4)	−0.010 (4)	0.003 (4)	0.016 (4)
C15	0.067 (5)	0.056 (5)	0.078 (5)	0.004 (4)	0.008 (4)	−0.014 (4)
Cl1	0.0749 (14)	0.0828 (16)	0.0814 (13)	0.0083 (11)	−0.0377 (11)	0.0094 (12)
Cl2	0.0537 (10)	0.0867 (14)	0.0685 (10)	−0.0146 (10)	−0.0044 (8)	0.0259 (10)
C9	0.033 (3)	0.060 (4)	0.049 (3)	0.003 (3)	−0.011 (2)	0.008 (3)
Cl3	0.0560 (10)	0.0825 (14)	0.0594 (9)	0.0141 (9)	−0.0025 (8)	−0.0122 (9)
Cl4	0.0789 (15)	0.0832 (17)	0.0793 (13)	−0.0066 (11)	−0.0410 (10)	−0.0038 (12)
C30	0.050 (3)	0.052 (4)	0.054 (3)	−0.007 (3)	−0.005 (3)	0.004 (3)
C8	0.084 (7)	0.100 (8)	0.129 (10)	−0.022 (7)	−0.043 (7)	0.072 (8)
C16	0.079 (5)	0.058 (5)	0.066 (4)	0.019 (4)	0.000 (4)	−0.011 (4)

### Geometric parameters (Å, °)

**Table:**

Sn1—C15	2.126 (8)	C29—C28	1.355 (14)
Sn1—C16	2.118 (7)	C29—C30	1.391 (12)
Sn1—N1	2.470 (5)	C29—H29	0.9300
Sn1—N2	2.468 (8)	C25—C26	1.466 (9)
Sn1—Cl1	2.5213 (19)	C25—H25	0.9300
Sn1—Cl2	2.4859 (19)	C26—N3	1.348 (8)
N1—C14	1.339 (8)	C26—C27	1.377 (9)
N1—C10	1.345 (8)	C17—C18	1.391 (9)
C10—C11	1.371 (9)	C18—C19	1.371 (9)
C10—C9	1.482 (8)	C18—C23	1.505 (10)
C13—C12	1.363 (14)	C21—C20	1.367 (11)
C13—C14	1.367 (12)	C21—H21	0.9300
C13—H13	0.9300	C23—H23A	0.9600
C4—C5	1.361 (12)	C23—H23B	0.9600
C4—C3	1.401 (12)	C23—H23C	0.9600
C4—C8	1.525 (11)	C19—C20	1.390 (11)
C11—C12	1.400 (11)	C19—H19	0.9300
C11—H11	0.9300	C28—C27	1.368 (11)
C7—C2	1.475 (12)	C28—H28	0.9300
C7—H7A	0.9600	C24—C20	1.500 (11)
C7—H7B	0.9600	C24—H24A	0.9600
C7—H7C	0.9600	C24—H24B	0.9600
C6—C1	1.371 (10)	C24—H24C	0.9600
C6—C5	1.382 (10)	C27—H27	0.9300
C6—H6	0.9300	C31—H31A	0.9600
C1—C2	1.393 (9)	C31—H31B	0.9600
C1—N2	1.441 (9)	C31—H31C	0.9600
C2—C3	1.384 (9)	N2—C9	1.254 (10)
C3—H3	0.9300	N3—C30	1.332 (8)
C12—H12	0.9300	C32—H32A	0.9600
C14—H14	0.9300	C32—H32B	0.9600
C5—H5	0.9300	C32—H32C	0.9600
Sn2—C31	2.124 (7)	C15—H15A	0.9600
Sn2—C32	2.130 (7)	C15—H15B	0.9600
Sn2—N3	2.456 (5)	C15—H15C	0.9600
Sn2—N4	2.449 (8)	C9—H9	0.9300
Sn2—Cl3	2.4908 (19)	C30—H30	0.9300
Sn2—Cl4	2.5170 (19)	C8—H8A	0.9600
N4—C25	1.277 (10)	C8—H8B	0.9600
N4—C17	1.456 (9)	C8—H8C	0.9600
C22—C17	1.365 (10)	C16—H16A	0.9600
C22—C21	1.399 (10)	C16—H16B	0.9600
C22—H22	0.9300	C16—H16C	0.9600
			
C16—Sn1—C15	170.2 (4)	N4—C25—C26	121.0 (6)
C16—Sn1—N2	91.4 (3)	N4—C25—H25	119.5
C15—Sn1—N2	82.5 (3)	C26—C25—H25	119.5
C16—Sn1—N1	82.9 (3)	N3—C26—C27	122.1 (6)
C15—Sn1—N1	87.8 (3)	N3—C26—C25	117.6 (5)
N2—Sn1—N1	68.3 (2)	C27—C26—C25	120.3 (6)
C16—Sn1—Cl2	95.9 (3)	C22—C17—C18	120.7 (6)
C15—Sn1—Cl2	92.2 (2)	C22—C17—N4	119.6 (6)
N2—Sn1—Cl2	95.58 (17)	C18—C17—N4	119.6 (6)
N1—Sn1—Cl2	163.73 (13)	C19—C18—C17	118.4 (6)
C16—Sn1—Cl1	91.6 (2)	C19—C18—C23	119.5 (6)
C15—Sn1—Cl1	92.0 (2)	C17—C18—C23	122.1 (6)
N2—Sn1—Cl1	162.27 (19)	C20—C21—C22	120.3 (7)
N1—Sn1—Cl1	94.76 (13)	C20—C21—H21	119.9
Cl2—Sn1—Cl1	101.49 (8)	C22—C21—H21	119.9
C14—N1—C10	117.3 (5)	C18—C23—H23A	109.5
C14—N1—Sn1	126.0 (4)	C18—C23—H23B	109.5
C10—N1—Sn1	116.3 (4)	H23A—C23—H23B	109.5
N1—C10—C11	123.1 (6)	C18—C23—H23C	109.5
N1—C10—C9	115.8 (5)	H23A—C23—H23C	109.5
C11—C10—C9	121.1 (6)	H23B—C23—H23C	109.5
C12—C13—C14	119.1 (8)	C18—C19—C20	122.0 (6)
C12—C13—H13	120.5	C18—C19—H19	119.0
C14—C13—H13	120.5	C20—C19—H19	119.0
C5—C4—C3	117.9 (7)	C29—C28—C27	119.2 (8)
C5—C4—C8	122.8 (10)	C29—C28—H28	120.4
C3—C4—C8	119.3 (9)	C27—C28—H28	120.4
C10—C11—C12	118.0 (7)	C20—C24—H24A	109.5
C10—C11—H11	121.0	C20—C24—H24B	109.5
C12—C11—H11	121.0	H24A—C24—H24B	109.5
C2—C7—H7A	109.5	C20—C24—H24C	109.5
C2—C7—H7B	109.5	H24A—C24—H24C	109.5
H7A—C7—H7B	109.5	H24B—C24—H24C	109.5
C2—C7—H7C	109.5	C28—C27—C26	119.6 (7)
H7A—C7—H7C	109.5	C28—C27—H27	120.2
H7B—C7—H7C	109.5	C26—C27—H27	120.2
C1—C6—C5	120.3 (7)	C21—C20—C19	118.7 (7)
C1—C6—H6	119.9	C21—C20—C24	120.5 (8)
C5—C6—H6	119.9	C19—C20—C24	120.8 (8)
C6—C1—C2	121.2 (6)	Sn2—C31—H31A	109.5
C6—C1—N2	119.4 (6)	Sn2—C31—H31B	109.5
C2—C1—N2	119.4 (6)	H31A—C31—H31B	109.5
C3—C2—C1	116.7 (7)	Sn2—C31—H31C	109.5
C3—C2—C7	121.1 (7)	H31A—C31—H31C	109.5
C1—C2—C7	122.2 (6)	H31B—C31—H31C	109.5
C2—C3—C4	122.9 (7)	C9—N2—C1	117.1 (7)
C2—C3—H3	118.5	C9—N2—Sn1	115.4 (5)
C4—C3—H3	118.5	C1—N2—Sn1	125.6 (5)
C13—C12—C11	119.2 (8)	C30—N3—C26	117.4 (6)
C13—C12—H12	120.4	C30—N3—Sn2	126.5 (4)
C11—C12—H12	120.4	C26—N3—Sn2	115.8 (4)
N1—C14—C13	123.3 (7)	Sn2—C32—H32A	109.5
N1—C14—H14	118.3	Sn2—C32—H32B	109.5
C13—C14—H14	118.3	H32A—C32—H32B	109.5
C4—C5—C6	120.9 (8)	Sn2—C32—H32C	109.5
C4—C5—H5	119.5	H32A—C32—H32C	109.5
C6—C5—H5	119.5	H32B—C32—H32C	109.5
C31—Sn2—C32	171.4 (3)	Sn1—C15—H15A	109.5
C31—Sn2—N4	83.1 (3)	Sn1—C15—H15B	109.5
C32—Sn2—N4	92.0 (3)	H15A—C15—H15B	109.5
C31—Sn2—N3	87.4 (3)	Sn1—C15—H15C	109.5
C32—Sn2—N3	84.2 (3)	H15A—C15—H15C	109.5
N4—Sn2—N3	68.4 (2)	H15B—C15—H15C	109.5
C31—Sn2—Cl3	91.8 (2)	N2—C9—C10	123.4 (6)
C32—Sn2—Cl3	95.7 (2)	N2—C9—H9	118.3
N4—Sn2—Cl3	95.82 (18)	C10—C9—H9	118.3
N3—Sn2—Cl3	164.18 (12)	N3—C30—C29	122.9 (7)
C31—Sn2—Cl4	91.7 (3)	N3—C30—H30	118.5
C32—Sn2—Cl4	90.9 (2)	C29—C30—H30	118.5
N4—Sn2—Cl4	162.1 (2)	C4—C8—H8A	109.5
N3—Sn2—Cl4	94.36 (13)	C4—C8—H8B	109.5
Cl3—Sn2—Cl4	101.46 (8)	H8A—C8—H8B	109.5
C25—N4—C17	115.7 (7)	C4—C8—H8C	109.5
C25—N4—Sn2	116.6 (5)	H8A—C8—H8C	109.5
C17—N4—Sn2	125.9 (5)	H8B—C8—H8C	109.5
C17—C22—C21	119.9 (7)	Sn1—C16—H16A	109.5
C17—C22—H22	120.1	Sn1—C16—H16B	109.5
C21—C22—H22	120.1	H16A—C16—H16B	109.5
C28—C29—C30	118.8 (8)	Sn1—C16—H16C	109.5
C28—C29—H29	120.6	H16A—C16—H16C	109.5
C30—C29—H29	120.6	H16B—C16—H16C	109.5
			
C16—Sn1—N1—C14	−80.3 (6)	C25—N4—C17—C18	119.1 (8)
C15—Sn1—N1—C14	102.6 (5)	Sn2—N4—C17—C18	−76.7 (8)
N2—Sn1—N1—C14	−174.6 (6)	C22—C17—C18—C19	0.3 (10)
Cl2—Sn1—N1—C14	−167.1 (4)	N4—C17—C18—C19	178.3 (6)
Cl1—Sn1—N1—C14	10.8 (5)	C22—C17—C18—C23	179.6 (7)
C16—Sn1—N1—C10	93.0 (5)	N4—C17—C18—C23	−2.4 (10)
C15—Sn1—N1—C10	−84.1 (5)	C17—C22—C21—C20	0.9 (13)
N2—Sn1—N1—C10	−1.3 (4)	C17—C18—C19—C20	1.7 (9)
Cl2—Sn1—N1—C10	6.1 (8)	C23—C18—C19—C20	−177.6 (7)
Cl1—Sn1—N1—C10	−176.0 (4)	C30—C29—C28—C27	0.8 (13)
C14—N1—C10—C11	−0.6 (9)	C29—C28—C27—C26	−0.6 (12)
Sn1—N1—C10—C11	−174.4 (5)	N3—C26—C27—C28	0.8 (11)
C14—N1—C10—C9	179.5 (5)	C25—C26—C27—C28	−179.7 (7)
Sn1—N1—C10—C9	5.6 (7)	C22—C21—C20—C19	1.0 (12)
N1—C10—C11—C12	1.3 (10)	C22—C21—C20—C24	179.6 (8)
C9—C10—C11—C12	−178.8 (7)	C18—C19—C20—C21	−2.4 (11)
C5—C6—C1—C2	−0.7 (11)	C18—C19—C20—C24	179.1 (7)
C5—C6—C1—N2	−179.6 (7)	C6—C1—N2—C9	−63.2 (10)
C6—C1—C2—C3	1.8 (10)	C2—C1—N2—C9	117.8 (8)
N2—C1—C2—C3	−179.3 (6)	C6—C1—N2—Sn1	100.3 (7)
C6—C1—C2—C7	−179.2 (8)	C2—C1—N2—Sn1	−78.7 (8)
N2—C1—C2—C7	−0.3 (11)	C16—Sn1—N2—C9	−85.7 (6)
C1—C2—C3—C4	−2.9 (10)	C15—Sn1—N2—C9	86.7 (6)
C7—C2—C3—C4	178.1 (8)	N1—Sn1—N2—C9	−3.9 (5)
C5—C4—C3—C2	2.8 (11)	Cl2—Sn1—N2—C9	178.2 (6)
C8—C4—C3—C2	−177.3 (8)	Cl1—Sn1—N2—C9	13.9 (10)
C14—C13—C12—C11	1.2 (13)	C16—Sn1—N2—C1	110.6 (6)
C10—C11—C12—C13	−1.6 (12)	C15—Sn1—N2—C1	−77.0 (6)
C10—N1—C14—C13	0.2 (10)	N1—Sn1—N2—C1	−167.6 (7)
Sn1—N1—C14—C13	173.4 (6)	Cl2—Sn1—N2—C1	14.5 (6)
C12—C13—C14—N1	−0.5 (12)	Cl1—Sn1—N2—C1	−149.8 (5)
C3—C4—C5—C6	−1.6 (12)	C27—C26—N3—C30	−1.2 (9)
C8—C4—C5—C6	178.6 (8)	C25—C26—N3—C30	179.3 (5)
C1—C6—C5—C4	0.6 (12)	C27—C26—N3—Sn2	−175.2 (5)
C31—Sn2—N4—C25	85.9 (6)	C25—C26—N3—Sn2	5.4 (7)
C32—Sn2—N4—C25	−87.0 (6)	C31—Sn2—N3—C30	102.2 (5)
N3—Sn2—N4—C25	−4.1 (5)	C32—Sn2—N3—C30	−79.8 (5)
Cl3—Sn2—N4—C25	177.0 (6)	N4—Sn2—N3—C30	−174.3 (6)
Cl4—Sn2—N4—C25	12.1 (10)	Cl3—Sn2—N3—C30	−170.2 (4)
C31—Sn2—N4—C17	−78.2 (6)	Cl4—Sn2—N3—C30	10.6 (5)
C32—Sn2—N4—C17	108.9 (6)	C31—Sn2—N3—C26	−84.5 (5)
N3—Sn2—N4—C17	−168.2 (7)	C32—Sn2—N3—C26	93.5 (5)
Cl3—Sn2—N4—C17	12.9 (6)	N4—Sn2—N3—C26	−1.0 (4)
Cl4—Sn2—N4—C17	−152.0 (5)	Cl3—Sn2—N3—C26	3.1 (8)
C17—N4—C25—C26	174.4 (6)	Cl4—Sn2—N3—C26	−176.1 (4)
Sn2—N4—C25—C26	8.7 (9)	C1—N2—C9—C10	174.0 (6)
N4—C25—C26—N3	−9.7 (10)	Sn1—N2—C9—C10	8.8 (9)
N4—C25—C26—C27	170.8 (7)	N1—C10—C9—N2	−10.1 (10)
C21—C22—C17—C18	−1.6 (11)	C11—C10—C9—N2	169.9 (7)
C21—C22—C17—N4	−179.6 (7)	C26—N3—C30—C29	1.5 (10)
C25—N4—C17—C22	−62.9 (10)	Sn2—N3—C30—C29	174.7 (5)
Sn2—N4—C17—C22	101.3 (8)	C28—C29—C30—N3	−1.3 (12)

### Hydrogen-bond geometry (Å, °)

**Table:**

*D*—H···*A*	*D*—H	H···*A*	*D*···*A*	*D*—H···*A*
C9—H9···Cl2^i^	0.93	2.73	3.608 (6)	157
C25—H25···Cl3^ii^	0.93	2.70	3.570 (7)	155

Symmetry codes: (i) *x*+1/2, −*y*+3/2, *z*; (ii) *x*−1/2, −*y*+1/2, *z*.
